# Why do we forget?—A mixed-method investigation of reasons for everyday prospective memory failures

**DOI:** 10.1186/s41235-026-00737-7

**Published:** 2026-06-05

**Authors:** Maximilian Haas, Alexandra Hering, Chiara Scarampi, Corinna S. Martarelli

**Affiliations:** 1 Faculty of Psychology, UniDistance Suisse, Schinerstrasse 18, Brig, Switzerland; 2 Faculty of Psychology and Educational Sciences, University of Geneva, Geneva, Switzerland; 3 Department of Developmental Psychology, Tilburg School of Social and Behavioral Sciences, Tilburg University, Tilburg, The Netherlands; 4 Center for the Interdisciplinary Study of Gerontology and Vulnerability, University of Geneva, Geneva, Switzerland

**Keywords:** Prospective memory, Intentions, Everyday memory failures, Daily diary, Ambulatory assessment

## Abstract

Prospective memory research often assumes that unfulfilled intentions reflect memory failures, but the reasons behind self-reported daily intention non-completion remain underexplored. In this study, we investigated the relative contribution of forgetting versus other factors to unfulfilled daily intentions and examined how intention-related characteristics predict the type of non-fulfillment. A total of 102 distance-learning students completed smartphone-based intention diaries across two five-day periods, reporting on 1823 non-routine next-day intentions and providing ratings of importance, perceived difficulty, memory confidence, and reminder use. Mixed-effects multinomial logistic regression revealed that memory confidence and perceived difficulty reliably distinguished non-forgetting reasons, whereas perceived importance had no effect. Reminder use was selectively associated with external causes rather than forgetting. Cross-validation with a publicly available dataset (Scott & Gilbert, 2024; *N* = 409 intentions) largely replicated these patterns. Overall, within the present sampling frame, forgetting accounted for only a minority of self-reported non-fulfillments (~ 10–14%), suggesting that situational and motivational factors, rather than memory failure, are primary drivers of non-completion in daily life. These findings challenge the assumption that unfulfilled intentions in naturalistic settings are predominantly due to forgetting and highlight the importance of considering multiple psychological and contextual aspects in prospective memory research.

## Significance statement

It is commonly assumed that failing to follow through on daily intentions, like missing an appointment or forgetting a task, primarily reflects memory problems. However, in our specific sampling context (self-assigned, non-routine next-day intentions from a volunteer student pool), most self-reported non-fulfillments were attributed to changing priorities, lack of time, or low motivation rather than to forgetting per se. These findings can help people and organizations design better strategies and support for accomplishing daily goals, focusing on addressing practical and motivational barriers rather than only trying to improve memory.

## Introduction

Prospective memory (PM), the ability to remember to execute intended actions in the future, plays a central role in managing everyday life (Einstein & McDaniel, 1990; Kliegel et al., 2016). Whether it is taking medication, calling a friend, or submitting important documents, our capacity to act on future intentions is essential to functional independence and effective self-regulation in daily routines, especially in older age (e.g., Brinkhof et al., 2024; Hering et al., 2018; Woods et al., 2012). Yet, PM is prone to error and previous research has shown that about half of all daily cognitive failures concern future intentions (e.g., Crovitz & Daniel, 1984; Haas et al., 2020; Niedźwieńska et al., 2020). Consequently, a substantial body of research has sought to understand why we fail our daily intentions, identifying different cognitive, emotional, and contextual factors that contribute to prospective lapses in everyday life.

### Forgetting and non-fulfillment

Over the past decade, naturalistic intention diary studies and ambulatory assessment methods have advanced the ecological validity of PM research by capturing self-assigned intentions in real-life contexts (cf. Rummel & Kvavilashvili, 2019). Typically, these studies focused on the frequency and predictors of fulfilled versus unfulfilled intentions. However, a critical yet largely overlooked assumption underlies this approach: When an individual reports that a planned action was not completed, the failure is often interpreted as a memory lapse—meaning that non-completion results from forgetting. However, this assumption is rarely tested. While experimental studies have manipulated factors such as delay intervals, cue salience, or multitasking to investigate the occurrence of PM errors (e.g., Einstein et al., 2005; Matos et al., 2020; Scullin et al., 2012), very few investigations have asked participants to report why they failed the accomplishment of a specific intention in an everyday context. This conflation overlooks an important distinction: While some instances of non-fulfillment indeed reflect forgetting, others may represent intentional decisions to abandon or postpone an intention.

Empirical work supports this distinction. In one of the earliest attempts, Maylor (1990) documented spontaneous reasons provided by participants following a call-back task. For the reported memory failures that occurred, the most frequently cited causes were being absorbed in other activities and unexpected changes in routine (i.e., distractions)—disruptions that reflect failure of the prospective component of PM (i.e., forgetting *that/when* something needed to be done) but not necessarily represent forgetting in a more mnemonic sense due to a failure in the retrospective component of PM (i.e., forgetting *what* needed to be done; McDaniel & Einstein, 2000). Extending this perspective, Marsh et al. (1998) found that in a series of naturalistic experiments with a total of 405 undergraduate students, participants most often reported having deliberately reprioritized their plans, while genuine forgetting accounted for only a small portion of non-completion (~ 13%). This underscores that successful PM comprises more than merely memory abilities, but rather a multidimensional set of factors. Inter- and intraindividual differences in metacognition, motivation, task meaningfulness, and executive processes to reprioritize intentions according to demands of everyday life, for example, may determine how individuals accomplish self-assigned intentions (see also Anderson et al., 2017; Dismukes, 2012; Roche et al., 2007). Recent advances in naturalistic PM research have built on this foundation. Rummel et al. (2023), combining diary methods and ecological memory assessment in a community sample, found that perceived intention importance, the use of external memory aids, delay interval, and conscientiousness were the most informative predictors of everyday intention fulfillment. These findings underscore that real-world PM is embedded in a broader motivational and personality context. Faustmann and Altgassen (2025) similarly demonstrated, using an experience sampling approach across a three-day period, that motivational factors moderate PM task execution in everyday life even after controlling for cognitive abilities, which provides further evidence that the ecology of PM is irreducible to a memory-only account. Converging evidence from research on goal pursuit and self-regulation further supports this view. Drawing on results from experience sampling studies, Hofmann and colleagues (2012) documented that everyday intention non-fulfillment is frequently driven by motivational conflict and competing desires rather than by any failure to remember what was planned. In their data, desires conflicting with other goals routinely prevented enactment. Research on goal pursuit and self-regulation has thus long recognized that the gap between intention and action reflects dynamic trade-offs among competing motivations, contextual demands, and volitional resources (cf. Baumeister et al., 2016; Gollwitzer & Sheeran, 2006); a perspective that PM research has only more recently begun to engage with systematically.

Despite these early insights, self-reported reasons for naturally occurring PM failures have received surprisingly little systematic attention. While some recent works have begun to classify and quantify the consequences of everyday memory lapses (e.g., Niedźwieńska et al., 2020), most studies using intention diary methods or naturalistic PM paradigms that track intention outcomes remain silent on whether missed intentions were forgotten or purposefully abandoned (e.g., Schnitzspahn et al., 2020; but see Niedźwieńska & Barzykowski, 2012–Study 2; Schnitzspahn et al., 2016). It is important at this point to distinguish between two methodological traditions that have been used to study everyday memory failures. In memory-failure diary studies, participants are explicitly instructed to record instances they themselves experience as forgetting either as they occur or retrospectively (e.g., Crovitz & Daniel, 1984; Laughland & Kvavilashvili, 2018—Study 3; Haas et al., 2020; Niedźwieńska et al., 2020). In these studies, instances of non-completion due to changing circumstances or deliberate reprioritization would not typically be perceived by the participant as a memory failure and would therefore not be reported. Although these studies have documented a prevalence of prospective over retrospective failure types (but see Unsworth et al., 2012), they do not—and do not aim to—estimate the relative prevalence of forgetting versus other reasons for intention non-fulfillment. By contrast, intention diary approaches, including the present study, prospectively track self-assigned intentions and subsequently assess their fulfillment and, where relevant, the reason for non-completion (e.g., Ellis & Nimmo-Smith, 1993). It is within this approach that the assumption that non-completion reflects forgetting has gone largely untested. As a result, the field risks overstating the role of forgetting and underestimating the influence of motivational, contextual, or circumstantial factors. This oversight has methodological as well as theoretical implications. If forgetting is not the primary reason for non-execution, then interpreting intention non-fulfillment as a memory failure may misrepresent the phenomenon under study. Moreover, it masks the complexity of goal management in everyday life, which often involves dynamic trade-offs between competing tasks, fluctuating motivation, and unforeseen constraints. Understanding *why* intentions are not fulfilled requires going beyond dichotomous outcome coding and toward a more fine-grained account of the subjective and situational contexts in which these non-fulfillments occur.

### Intention-related characteristics

Beyond identifying reasons for non-fulfillment, understanding which characteristics of an intention make them more likely to not be executed for specific reasons adds an important layer of information. Prior work has shown that the perceived importance, perceived difficulty, reminder use, and confidence in remembering an intention are associated with execution rates (e.g., Ihle et al., 2012; Marsh et al., 1998; Scarampi et al., 2024; Schnitzspahn et al., 2020), but these assessments have not been linked directly to the reason for non-fulfillment. For instance, it remains unclear whether intentions that are truly forgotten differ systematically in subjective characteristics from those that are consciously postponed, rescheduled, or abandoned due to external interference. Investigating these associations may shed light on how people evaluate and monitor their intentions, and whether certain types of intentions are more vulnerable to specific types of non-completion.

### Study goals and hypotheses

The present study aimed to address this gap by combining quantitative and qualitative approaches to investigate reasons for non-fulfillment of everyday PM intentions. In a first step, we aimed to develop a comprehensive and structured typology of non-fulfillment reasons by synthesizing prior literature, researcher expertise, and artificial intelligence-assisted clustering of open-text responses from a previous dataset. The resulting classification was then integrated into a daily diary in which participants recorded their everyday intentions and, in cases of non-fulfillment, selected the reason that best described why the task had not been completed.

In addition, participants rated each intention at the time of entry in terms of its subjective importance, perceived difficulty of completing the intended task, confidence in remembering, and also retrospectively reported the use of external reminders after planned execution. These intention-related ratings allowed us to examine how individual perceptions relate to both overall intention fulfillment and the specific self-reported reasons for non-fulfillment. In particular, we were interested in whether intentions reported as truly forgotten differ in these features from intentions that were not completed for other reasons, such as rescheduling or lack of motivation.

Finally, to examine the robustness of our findings and enhance generalizability, we sought to replicate key patterns in a second dataset that had been made publicly available during our data collection (cf. Scott & Gilbert, 2024). This dataset used a similar online-diary method and included information on both intention outcomes and subjective ratings. However, the specific analyses reported here were not conducted by the original authors. We aimed to cross-validate both the relative frequency of non-fulfillment reasons and the association between intention-related factors and execution success. By disentangling forgetting from other forms of non-fulfillment, the present study seeks to challenge common assumptions in PM research and contribute to a more accurate and ecologically grounded understanding of why it can be challenging to execute our daily intentions.

## Methods

### Participants and study procedure

Data were collected from 102 adult participants (*M* = 37.26 years, *SD* = 8.50, age range 19–62 years, 84 women) between November 2023 and April 2024 in Switzerland. Participants were recruited from an internal pool of volunteers enrolled in the undergraduate psychology program. After providing informed consent, participants completed a 30-day ambulatory assessment using the smartphone application *movisensXS* version 1.5.23 (movisens GmbH, Karlsruhe, Germany). Following a baseline survey that included sociodemographic information and a set of questionnaires (not reported here), participants recorded self-generated daily intentions in a memory diary and reflected on their (non-)fulfillment of these intentions throughout the study period (see below for details of the task and the diary procedure).

### Study material

As part of a larger project, additional measures were collected beyond the focus of the present study. Below, we describe only the materials and procedures relevant to the current investigation. For additional details on the study protocol and remaining variables, please refer to the material available on the Open Science Framework (OSF) at osf.io/tu2dx.

#### Development of reason categories

To identify reasons for non-completion of daily intentions, we employed a three-step iterative approach combining human judgment with artificial intelligence-based assistance. First, the authors and a trained research assistant independently generated a broad set of possible reasons for failures in everyday PM, based on personal experience, literature knowledge, and intuitive reflection. Second, we used ChatGPT-4 (OpenAI, 2025) through the web app [OCT 31, 2023] to extend this collection, yielding reasons such as lack of time or motivation, poor prioritization, external circumstances, and health-related problems (see OSF for the full list of generated reasons and the specific prompt used). Third, we analyzed 251 open-text responses on intention non-fulfillment from a prior study (Haas et al., 2022) with ChatGPT-4, asking it to cluster the explanations into meaningful categories (see OSF for the prompts used and interim results). The most frequently identified themes included ‘reprioritization or other commitments’ (24.9%), ‘lack of time’ (22.2%), ‘motivation/fatigue’ (15.3%), and ‘weather/external conditions or technical problems’ (15%); also see^1^. These categories then served as the basis for our final classification, integrated with prior literature (Marsh et al., 1998; Maylor, 1990; Schnitzspahn et al., 2016).

For the final, human-audited classification, we retained the following six major recurring reasons: (1) ‘Forgotten,’ (2) ‘Change in priorities/Rescheduled,’ (3) ‘Lack of time,’ (4) ‘Fatigue/Lack of motivation,’ (5) ‘Medical reason/Poor health,’ and (6) ‘External causes’ (e.g., bad weather, technical issues, and got canceled). Being aware that these categories cover a majority but not all possible reasons, participants were given the possibility to select an open-ended option (7) ‘Other reason.’

#### Everyday intentions

*Intention Diary* Twice during the 30-day study period (i.e., with a two-week interval), participants completed a smartphone-based intention diary over five days each (for a similar procedure, see Haas et al., 2022; Ihle et al., 2012). Every evening, participants received a push notification prompting them to list two of their personal intentions/non-routine tasks for the following day. On the subsequent evening, participants were presented with their previously entered intentions and asked to indicate whether each intention had been completed. For intentions that were not fulfilled, participants were asked to select a reason from the list of seven categories described above.

In terms of domain, the intentions reported in the present sample covered a broad range, with the most prevalent categories being organizational/administrative or housekeeping tasks (31.05%; e.g., paying a bill, buying something at the store, watering the plants), work- or study-related tasks (26.44%; e.g., team meetings, preparing a presentation, or handing in a report), social commitments (17.66%; e.g., calling/texting/meeting a family member or a friend), leisure activities (15.74%; e.g., going to the cinema or a concert, attending a dancing class, getting a haircut), and health-related intentions (4.88%; e.g., appointment with doctor/dentist, picking up treatment at the pharmacy). In terms of temporal structure, the reported intentions were almost exclusively related to time-based rather than event-based plans, consistent with prior diary research indicating that self-assigned everyday intentions are predominantly organized around specific times or time windows rather than environmental cues (e.g., Schnitzspahn et al., 2020). Representative qualitative data examples of each domain are shown in Appendix Table 3.

*Intention-Related Ratings* To explore factors associated with the success or failure of daily intentions, participants provided additional ratings for each intention at the time of entry. These variables were selected based on prior literature linking intention characteristics to PM performance. Specifically, participants rated (a) the importance of completing the intention (1 = not important at all; 5 = very important), (b) confidence in remembering to complete the intention (1 = not confident at all; 5 = very confident), and (c) its perceived difficulty (1 = not difficult at all; 5 = very difficult). In addition, after the planned time of execution, participants were asked to state whether they had used an external reminder (yes/no) to help remember the intention, independently of whether the intention had actually been fulfilled or not.

### Statistical analyses and cross-validation of results

All statistical analyses were conducted in R (version 4.4.0; R Core Team, 2025). First, we performed descriptive analyses to investigate the extent to which self-reported forgetting accounts for daily intention failures compared to other subjective reasons for failure. Second, we computed mixed-effects multilevel logistic regression (using the glmer function from the R package lme4 [version 1.1–35.3]; Bates et al., 2015) to inspect the effect of each of the item-related characteristics on general intention fulfillment. The dichotomous outcome variable (i.e., intention fulfillment) was modeled using a binomial error distribution with a logit link function. Continuous model predictors (i.e., importance, confidence, and perceived task difficulty) were standardized through grand-mean centering and scaling to unit variance prior to analysis, yielding coefficients that can be interpreted as change in log-odds. Reminder use was included as a categorial fixed effect with two levels, treating the ‘no reminder’ condition as the reference category. Thus, the coefficient for reminder use represents the difference in log-odds of checking behavior between intentions for which individuals did and did not use a reminder. Random intercepts were included to account for the nesting of multiple intentions within participants.

To examine specific subjective reasons for non-completion, we then performed a mixed-effects multinomial logistic regression (using the R package mgcv [version 1.9–1.]; Wood, 2015), assessing how subjective intention-related characteristics relate to specific non-fulfillment categories. For this analysis, we only focused on the subset of intentions for which participants indicated non-completion. Moreover, cases reported as ‘other reason’ were excluded to ensure interpretability of category-specific effects. For the multinomial model, ‘forgetting’ was set as the reference baseline to allow for meaningful interpretation of contrasts. As above, continuous predictors were standardized through grand-mean centering and scaling; and participant-specific random intercepts were included to account for individual differences in overall response tendencies and repeated observations nested within participants. The multinomial model was implemented using a list of parallel sub-models (i.e., one representing the baseline category and one representing each of the remaining *K* = 5 non-baseline reason categories); each sharing an identical set of predictors. Model fitting was performed using restricted maximum likelihood (REML).

*Cross-Validation Dataset* In addition to these analyses applied to our own data, we attempted to replicate and cross-validate our findings with a dataset that was made publicly available toward the end of our data collection (Scott & Gilbert, 2024). In their online survey study conducted on Prolific, 112 adult participants reported upcoming plans, including non-routine activities, for the following 3 days (Wave 1) and their subsequent fulfillment 4–5 days later (Wave 2). The dataset includes plan-level importance, confidence, reminder use, and self-reported reasons for non-fulfillment (with a coarser taxonomy of non-fulfillment reasons than the classification developed in the present study). The participants of their study were *M* = 35.16 years old (*SD* = 12.64; range: 19–70 years), predominantly white (73.22%), and the majority described their situation as (full-/part-time/self-) employment (67.86%, *n* = 76) or as being a student (16.07%, *n* = 18). While this dataset includes participants’ reported reasons for unfulfilled intentions, these were not the primary focus in the original publication. Analytical approaches were identical for the replication procedure (i.e., descriptive analyses of non-fulfillment categories and inferential approaches to investigate differences in intention-related factors). Only intention-related factors common to both datasets were analyzed (i.e., importance, confidence, and reminder use; as perceived task difficulty was not part of the replication dataset).

## Open practices statement

This study’s design, hypotheses, and analytic plans were not preregistered. We report our sample size, and describe all data exclusions (if any), manipulations, and all measures relevant to the present study. De-identified, pre-processed data and annotated analytical codes underlying the present analyses are openly available at osf.io/tu2dx. Additionally, the classification-drafting interaction with the ChatGPT web app [OCT 31, 2023], model version GPT-4, is reported and archived on the OSF. The ChatGPT web app does not expose sampling parameters (e.g., temperature, top-p, seed), and these cannot be recovered post hoc. The target sample size for this study was set at *N* = 100 following recommendations in the literature (e.g., Bolger et al., 2012; Fritz et al., 2024). Simulation-based computations by Arend and Schäfer (2019) indicate a minimal detectable effect size of.14 for *n* = 8 repeated measures in a sample size of *N* = 100 individuals, highlighting adequate power for detecting small to medium effects in the present study design.

## Results

### Reasons for non-fulfillment

In total, the 102 participants listed *N* = 1823 intentions, of which 565 (i.e., 30.9%) were reported as not accomplished. When looking at the specific self-reported reasons provided for not accomplishing PM intentions, subjective forgetting about the intention was not reported as the main reason (see Table 1). In fact, it appeared that intentions were not completed at the planned moment more frequently because of other external and internal causes, such as the need to reschedule initial plans or due to a lack of time or motivation. Actual forgetting was only reported as the reason for non-completion of about one in ten daily intentions.

**Table 1:** Frequency of reasons for unfulfilled daily intentions

Reason	*n*	% of total
Change in priorities/Rescheduled	190	33.63
Lack of time	97	17.17
Fatigue/Lack of motivation	86	15.22
Other reason (unspecified)	60	10.62
Forgetting	54	9.56
External cause (e.g., bad weather, technical issues, …)	50	8.85
Medical reasons/Poor health	28	4.96

Table of absolute and relative reporting rates of each failure reason for all 565 intentions of the present dataset. Reasons are ordered from the highest to the lowest frequency

### Intention-ratings and intention fulfillment

**Fig. 1:**
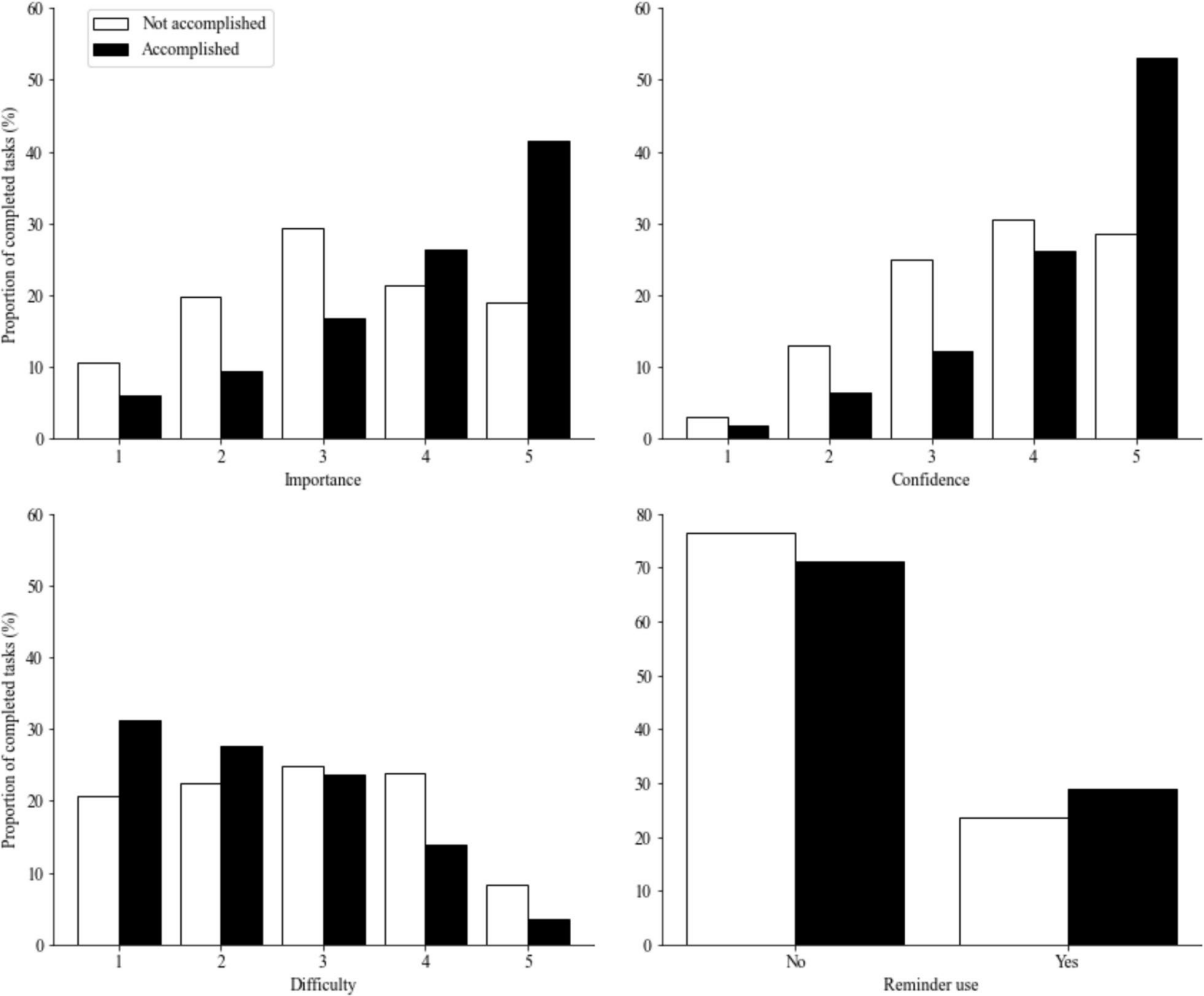
Rating-specific proportions of intention fulfillment. *Note* Bars represent the proportion of intentions reported as fulfilled (black) or non-fulfilled (white) for each point on the 5-point Likert scale in the respective intention-rating. Bars of the same color in each quadrant sum to 100%

Figure 1 illustrates the proportions of accomplished versus non-fulfilled intentions for each of the specific item-related characteristics. In line with previous research, results of the mixed-effect multilevel logistic regression revealed significant associations for several predictors. Importance ratings were positively associated with frequency of executed intentions (*B* = 0.625, *p* <.001, 95% CI [0.442, 0.808]; OR = 1.868), such that intentions considered as less important were less frequently accomplished. Subjective task difficulty ratings were negatively associated with frequency of intention execution (*B* = − 0.481, *p* <.001, 95% CI [− 0.665, − 0.335]; OR = 0.606), in the sense that intentions rated as more difficult were more frequently reported as not fulfilled. Furthermore, a significant association of intention execution was observed for memory confidence (*B* = 0.236, *p* =.005, 95% CI [0.072, 0.416]; OR = 1.276). Only intentions for which participants were most confident were executed more frequently, whereas all lower confidence ratings were associated with higher frequency of non-accomplishment. The reported use of reminders, however, was not significantly associated with intention accomplishment (*B* = −0.139, *p* =.396, 95% CI [− 0.579, 0.229]; OR = 0.839).

### Intention-ratings and reason categories

We estimated a mixed-effects multinomial logistic regression to examine whether intention-related characteristics (i.e., importance, perceived difficulty, confidence, and use of reminders) were associated with the reasons why certain intentions were not accomplished. The categorical outcome variable was the self-reported reason for non-fulfillment, with ‘forgetting’ serving as the reference category. Random intercepts for participants were included to account for the nesting of daily diary intentions within individuals. Table 2 shows the summary results.

**Table 2:** Relative risk ratios (RRR) of non-fulfillment reasons relative to forgetting

Reason (vs forgetting)	Importance	Confidence	Difficulty	Reminder use
Change in priorities/Rescheduling	0.679 *p* =.154	**1.968** *p* =.008	**1.846** *p* =.019	1.963 *p* =.331
Lack of time	1.007 *p* =.981	**2.498** *p* =.001	**2.109** *p* =.009	1.605 *p* =.525
Fatigue/Lack of motivation	0.728 *p* =.302	**1.798** *p* =.041	**2.087** *p* =.017	0.759 *p* =.742
Medical reasons/Poor health	0.766 *p* =.572	**7.614** *p* =.002	1.893 *p* =.166	2.384 *p* =.425
External causes	0.923 *p* =.823	**2.072** *p* =.046	1.232 *p* =.568	**9.963** *p* =.006

RRR > 1 indicates higher odds of the listed reason relative to ‘forgetting’ for a 1 SD increase in the predictor (or change in reminder use ‘yes vs no,’ respectively). Significant results are marked in bold font

#### Importance

Perceived importance did not significantly predict whether intentions were not accomplished due to forgetting compared to any other reason (all *p*s >.15).

#### Perceived difficulty

 In contrast, greater perceived difficulty was predictive of certain non-forgetting reasons. Specifically, higher reported subjective difficulty of the task was associated with a higher likelihood of citing competing demands (*B* = 0.613, *p* =.019, 95% CI [0.103, 1.124]; OR = 1.968), lack of time (*B* = 0.746, *p* =.009, 95% CI [0.189, 1.307]; OR = 2.109), and motivational factors (*B* = 0.732, *p* =.013, 95% CI [0.155, 1.308]; OR = 2.087) over forgetting. In other words, intentions that were perceived as more difficult were more likely to be attributed to situational or motivational challenges rather than to memory failure.

#### Confidence

Higher ratings of memory confidence reliably distinguished forgetting from other reasons of non-completion. In fact, each one-unit increase in confidence was associated with higher odds of reporting competing demands rather than forgetting (*B* = 0.677,* p* =.008, 95% CI [0.177, 1.175]; OR = 1.968), greater likelihood of citing lack of time (*B* = 0.915,* p* =.001, 95% CI [0.350, 1.479]; OR = 2.498) or motivation (*B* = 0.587, *p* =.041, 95% CI [0.021, 1.143]; OR = 1.798), and of reporting health (*B* = 2.029, *p* =.002, 95% CI [0.736, 3.314]; OR = 7.614) or other external causes (*B* = 0.728, *p* =.046, 95% CI [0.097, 1.481]; OR = 2.072 relative to forgetting. Thus, non-fulfillment of intentions for which participants were more confident was less likely to be attributed to forgetting and more likely to be caused by other situational or internal causes.

#### Reminder use

Reminder use showed a selective effect. Using reminders was positively associated with reporting external causes compared to forgetting (*B* = 2.299, *p* =.006, 95% CI [0.522, 3.746]; OR = 9.963). This suggests that non-fulfillment of intentions for which individuals employed reminders was less likely attributed to mere forgetting but was more likely reported as caused by external barriers such as bad weather, technical difficulties, or cancelations.

### Cross-validation of findings

Additionally, we sought to cross-validate our findings with a dataset by Scott and Gilbert (2024) that is publicly available on the OSF (https://osf.io/cp6h8/). In their set of online-diary experiments with 112 adult participants conducted on Prolific, the authors also collected data on reasons for not fulfilling planned intentions, as well as ratings of importance and confidence (but not perceived task difficulty). Specifically, in a first survey, participants were asked to list upcoming plans for the following three days, including any non-routine activity or appointment (e.g., ‘Meet John,’ ‘Complete presentation,’ or ‘Catch train to X’). Each plan had to be rated for the importance of remembering it, the use of reminders, and the confidence in remembering to execute the plan. Four to five days after the initial listing, participants filled in a second survey, indicating whether the planned intention had been completed and, if unfulfilled, provided the reason from a set of options (e.g., ‘forgot about it,’ ‘forgot or lost necessary information,’ ‘waiting for a better opportunity,’ ‘something else was more important/favorable to do,’ ‘barely possible to perform the intention due to circumstances’).

The dataset contains *N* = 409 planned intentions, of which 131 have been reported as not fulfilled (32.0%). Although the spectrum of potential reasons was less detailed than in the present paper, findings revealed that ‘forgetting an intention’ was not the most frequent reason for non-fulfillment. Specifically, only 13.7% of all cases, participants forgot the intentions, which is broadly consistent with the range reported in the present study (9.56%).

Computing a multinomial logistic regression model revealed that confidence was a consistent predictor of failure reasons. In fact, intentions for which participants indicated higher memory confidence were more likely reported as non-fulfilled because of impossibility to enact (*B* = 0.867, *p* =.019, 95% CI [0.144, 1.591]; OR = 2.381), potential better opportunities to accomplish (*B* = 0.921, *p* =.015, 95% CI [0.176, 1.667]; OR = 2.512), and other important priorities (*B* = 0.872, *p* =.012, 95% CI [0.189, 1.554]; OR = 2.391) rather than being forgotten. However, importance (all *p*s >.147) and reminder use (all *p*s >.417) were not significantly associated with specific reasons relative to forgetting. Overall, the replication dataset largely reproduces the pattern from the main study: Memory confidence distinguishes non-forgetting reasons, and importance has no effect. Reminder use, however, was not significant in the replication data, possibly due to differences in sample characteristics or the range of reasons assessed.

## Discussion

The present study set out to investigate why people fail to follow through on their everyday intentions, drawing attention to the widely held implicit assumption in PM research that unfulfilled intentions necessarily reflect forgetting. Across two independent datasets, our findings provide converging evidence that self-reported forgetting accounts for only a minority of not executed intentions. Instead, individuals most frequently attributed non-fulfillment to situational and motivational reasons, such as competing demands (i.e., reprioritization), lack of time, low motivation, or external constraints. These results highlight the importance of moving beyond memory-centric accounts of PM failure toward a more comprehensive framework that acknowledges the interplay of cognitive, motivational, and contextual factors.

### Rethinking everyday PM failures

Over a 30-day period, we asked participants to record their daily intentions for two weeks and, in cases of non-fulfillment, indicate the reason that best described why the planned intention had not been completed. Our data, and the present results overall, underscore that non-completions recorded within the intention diary paradigm are not synonymous with memory lapses. Non-fulfillment, as captured by this method, is a broader outcome that comprises genuine forgetting alongside other reasons. In fact, actual forgetting constituted less than 10% of reported reasons for non-completion in the main dataset, with similar proportions emerging in the replication dataset. These numbers align with earlier work (e.g., Marsh et al., 1998; Maylor, 1990) suggesting that everyday intention non-fulfillment is most often caused by deliberate reprioritization or (internal and external) contextual barriers. These converging findings underscore that interpreting non-fulfillment in daily diaries or ecological study designs as evidence of forgetting may systematically misrepresent the phenomenon of naturalistic PM failures. This contrast with the memory-failure diary paradigm, which by design captures only those memory failures experienced as such and thus addresses a related but distinct question. From a theoretical perspective, this calls for integrating prospective remembering into broader models of everyday goal management, where both failures and the deliberate decision not to execute a planned intention (i.e., rational and adaptive response to changing circumstances) can arise at multiple stages, from encoding and planning to maintenance, retrieval, or execution (cf. Bayen et al., 2022; Kvavilashvili & Ellis, 1996; Zuber & Kliegel, 2020). Future research might disentangle the role of the prospective and the retrospective component of daily PM intentions at each of these stages in a more systematic manner in order to determine their contribution to reasons for non-fulfillment.

### Intention-related characteristics and specific reasons

A novel contribution of this study is the integration of subjective ratings of intentions (i.e., task importance, perceived difficulty, confidence, and reminder use) with self-reported reasons for non-fulfillment. Our results showed that perceived difficulty and memory confidence significantly distinguished forgetting from other types of non-fulfillment. In fact, intentions perceived as more difficult were disproportionately associated with situational reasons such as reprioritization and lack of time, while higher confidence was linked with external causes rather than forgetting. The finding that higher confidence was not linked to self-reported forgetting may indicate good metacognitive calibration, in that participants who felt confident about remembering were in fact less likely to forget. Interestingly, perceived importance did not reliably predict reasons for intention non-fulfillment, although it strongly predicted overall PM success as reported in previous literature (e.g., Hering et al., 2014; Ihle et al., 2012; Niedźwieńska & Barzykowski, 2012; for a review see Walter & Meier, 2014). Reminder use in the main dataset—but not in the replication—showed a selective effect, in the sense that individuals who reported using reminders were less likely to attribute non-fulfillment to forgetting, but more likely to indicate external causes. Taken together, these findings suggest that subjective perceptions of intentions not only influence whether tasks are accomplished, but also how individuals interpret their failures when intentions are not fulfilled as planned.

### Limitations and future directions

Several limitations should be noted. First, the study relied on self-reports, which may be subject to biases such as limited introspective access, retrospective rationalization, or social desirability. Participants may not always be able to accurately distinguish forgetting from deliberate postponing, and attributions may reflect self-protective tendencies (e.g., social desirability). Yet, converging ranges of ‘forgetting’ as a subjective reason across multiple studies and datasets strengthen the robustness of our findings. Future research could address this limitation by using mixed-method approaches that combine self-report with objective behavioral or sensor-based measures of everyday intentions. Second, because our diary approach targeted non-routine next-day intentions, routine and habitual intentions (e.g., repeated medication intake, daily school pick-up, etc.) were intentionally under-sampled by design. Moreover, less salient or add-on intentions may often go underreported altogether. These characteristics can influence both completion rates and the distribution of non-fulfillment reasons, thus limiting generality to other types of intentions. Third, while our classification scheme was developed through a multi-step process combining previous literature, researcher expertise, and AI-assisted computational clustering, participants still used the ‘other’ category in about 10% of all non-fulfillments, indicating that additional or more fine-grained categories may potentially be needed. Future studies may gather a more important and more diverse dataset of daily intentions and non-fulfillment reasons to develop a classification scheme with even higher precision.

Furthermore, extending the present approach to a broader age range would be particularly informative. Established findings on the age-PM paradox indicate that older adults do not show higher rates of PM non-completion in naturalistic and diary-based PM; indeed, they often outperform younger adults on close-to-real-life PM tasks, likely reflecting age-related differences in various underlying factors such as motivation, time structure, and strategy use (e.g., Henry et al., 2004; Schnitzspahn et al., 2020). In addition, age-related differences in metacognitive monitoring may influence not only whether an intention is remembered, but also how its non-completion is subsequently interpreted and attributed (e.g., Cauvin et al., 2019a, 2019b; Scarampi et al., 2024). Younger adults, who tend to experience more competing demands, greater daily activity absorption, and higher perceived stress (e.g., Festini et al., 2019; Schnitzspahn et al., 2011), may be more likely to attribute non-fulfillments to goal-management factors such as reprioritization or lack of time. Older adults’ less frequent non-fulfillments, by contrast, might show a relatively higher proportion attributed to subjective forgetting—not because they forget more in absolute terms, but because the non-fulfillments that do occur are less dominated by scheduling and motivational influences that characterize younger adulthood. Whether older adults differ from younger adults in the pattern of subjective reasons they attribute to intention non-fulfillment—and whether attributional tendencies interact with the type or importance of the intentions—remains an open question for future diary research with broader age samples. It will also be essential to investigate how the distribution of reasons varies across task types (e.g., event- vs. time-based PM) and time horizons (i.e., short and long delay intervals). Finally, exploring how individual differences in motivation, emotion regulation, or executive functioning shape both intention-related characteristics and non-fulfillment attributions could shed further light on the mechanisms underlying everyday PM.

### Constraints on generality

Although the present sample extends previous research in PM by including middle-aged adults, some limitations of generalizability need to be noted. First, the study drew on a sample that was WEIRD (Western, educated, industrialized, rich and democratic; Henrich et al., 2010), and—although not specifically assessed—likely not clinically selected or ethnically diverse. In addition, participants were distance-learning students who balanced university coursework with regular (full-time) employment, therefore potentially introducing a bias toward individuals with higher self-discipline and established routines. While these sample characteristics might enhance real-world applicability compared to samples with full-time students, future research involving more diverse or potentially clinically relevant populations is needed. Although we observe similar results in the cross-validation sample, which was drawn from a broader pool of participants, this should be interpreted as convergent but not definitive evidence. Therefore, prevalence figures for reasons of non-fulfillment should not be generalized to other populations or intention types without caution.

## Conclusions

In conclusion, the current findings make two important methodological contributions with relevant conceptual implications. First, they showed the value of explicitly assessing the reasons for non-fulfillment rather than assuming or reducing it to forgetting by default. By documenting the reasons individuals themselves report for not fulfilling real-life intentions, we showed that failures are often rooted in contextual constraints, motivational challenges, or strategic reprioritization. These findings challenge the common practice of treating non-fulfillment as a direct proxy for memory lapses and call for a more elaborate, ecologically grounded understanding of PM. Second, our findings highlight the usefulness of combining intention-related ratings with reason categories to capture the different ways in which individuals experience and explain why they may not execute their intentions. Recognizing the diversity of reasons for everyday non-fulfillment not only enriches theoretical models of PM, as it reflects motivational trade-offs, contextual demands, and individual coping strategies, but it also brings PM research closer to everyday life, where successful goal management often involves flexible reprioritization rather than rigid adherence to initial plans. This can shape the practical implications for designing interventions that better support individuals in managing their future goals.

## Data Availability

This study’s design, hypotheses, and analytic plans were not preregistered. De-identified, pre-processed data and annotated analytical codes underlying the present analyses are openly available at osf.io/tu2dx.

## Appendix

See Table 3.

**Table 3:** Qualitative examples of daily intentions and their respective characteristics classified by domain

Domain	Importance	Difficulty	Confidence	Reminder use
Social (*n* = 322|20.81% unfulfilled)				
Calling a friend to ask how he is doing	2	1	1	1
Going to an exhibition with a friend*	3	2	4	1
Dinner plans	3	1	5	1
Visiting a friend at the hospital	5	3	4	0
Work-/Study-related (*n* = 482|34.33% unfulfilled)				
Write a job application	5	4	5	0
Prepare flash cards for study lesson	2	1	5	0
Appointment with a client	5	4	5	1
Send an email to management	3	2	4	1
Organizational/Housekeeping (*n* = 566|32.51% unfulfilled)				
Bring documents to the bank office	3	2	4	0
Send off the rental agreement	5	3	2	0
Get refund for a shirt that I bought	2	2	4	0
Get a new car insurance	1	1	3	1
Leisure (*n* = 287|34.84% unfulfilled)				
Go to the Tai Chi class	5	3	5	1
Stop by the jewelery store	1	1	5	0
Get a haircut	3	1	5	1
Go to an improv show	4	1	5	1
Health (*n* = 89|21.35% unfulfilled)				
Do my physio exercises	4	2	4	0
Vaccination appointment	4	1	4	1
Appointment with a doctor	2	3	2	1
Go to the pharmacy	2	2	4	0

^*^Intentions that specifically included another person in the description were coded as social intentions, even if the content would else be classified as another domain. Importance = self-rated importance of accomplishing the intention on that specific day, Likert scale 1–5 (higher values represent higher importance); Difficulty = perceived difficulty of accomplishing the intention, Likert scale 1–5 (higher values represent higher perceived difficulty); Confidence = subjective rating of confidence in remembering to accomplish the intention, Likert scale 1–5 (higher values represent higher confidence); Reminder use = retrospective indication of whether a reminder has been used (= 1) compared to no use of reminder (= 0) to remember the intention (irrespective of whether the intention was accomplished or not)

## Footnotes

1 We draw the reader’s attention to the fact that numerical values and proportions provided by the AI-assisted clustering may depend on the parameter specifications of the LLM used and are therefore not fully reproducible with different models or specifications (also see the Open Practices Statement in the Method section above for further details). As these outputs were used only to support the qualitative development of the taxonomy, they should be interpreted as approximative rather than definitive.
